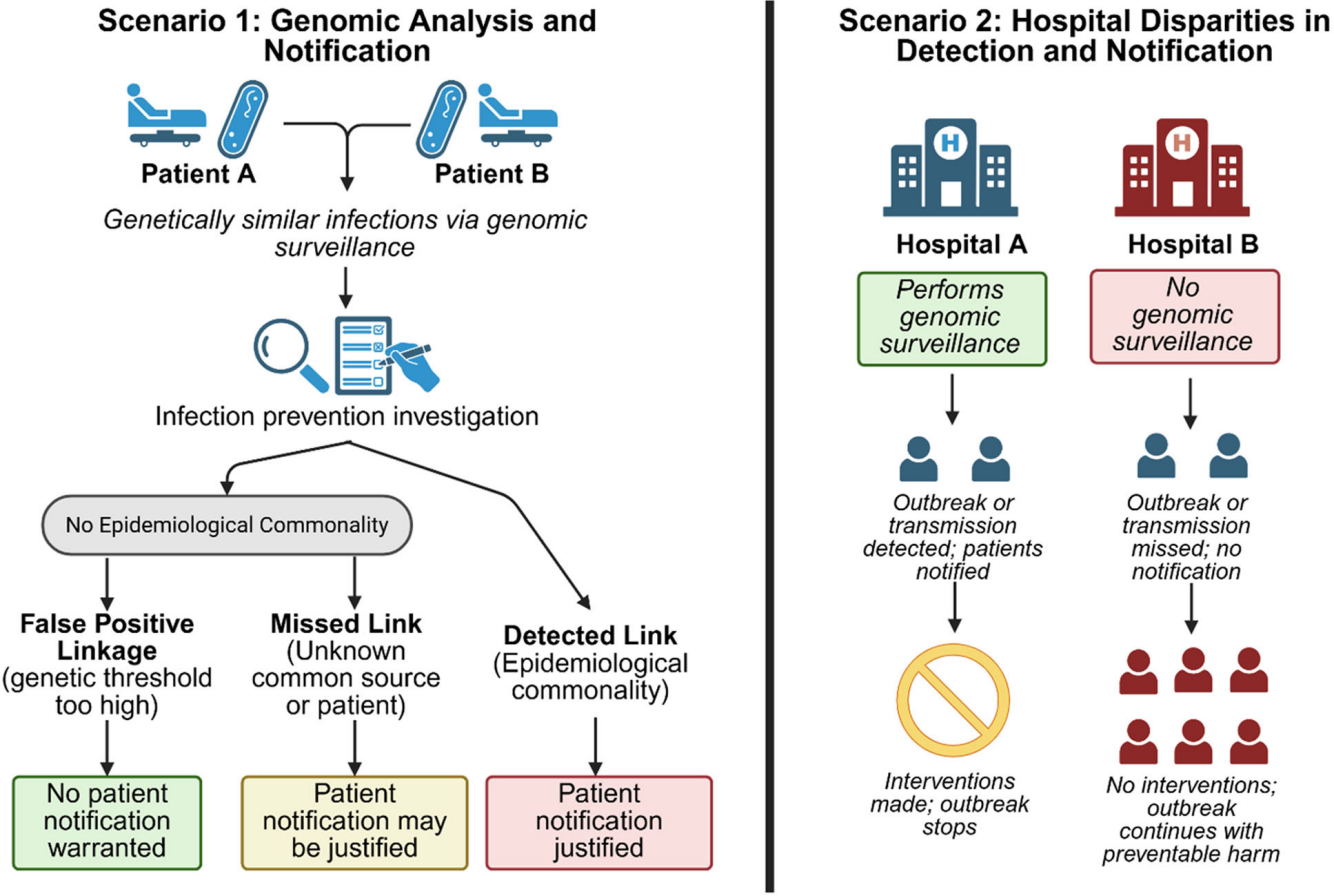# 310 The Role for Genomic Sequencing of Candidozyma auris in Healthcare Settings

**DOI:** 10.1017/ash.2026.10661

**Published:** 2026-06-23

**Authors:** Alexander Sundermann, Waleed Javaid, Lisa Parker

**Affiliations:** 1 University of Pittsburgh

## Abstract

**Background:** Genomic surveillance detects healthcare-associated outbreaks that are often missed by traditional methods, offering substantial benefits for infection prevention. However, its use introduces complex ethical dilemmas regarding patient notification and disparities in outbreak detection between institutions with and without genomic capabilities. **Methods:** We applied the Centers for Disease Control and Prevention's 2020 ethical considerations regarding patient notification and the Ethical Infection Prevention and Control (EIPAC) framework to two hypothetical scenarios involving genomic surveillance programs. Our analysis was informed by qualitative research reporting the types of harm experienced by patients across detected and undetected outbreaks. **Results:** We identified ethical justifications for patient notification when both genomic and epidemiologic evidence suggest transmission, as well as considerations militating for and against notification when epidemiologic evidence supporting genomic findings is absent (Figure). We also demonstrated ethical concerns arising from institutional disparities in genomic capability such that patients in hospitals without genomic surveillance may suffer preventable harm and remain uninformed due to lack of detection. A harms matrix illustrates physical, psychological, and ethical harms depending on detection and disclosure status. **Conclusion:** Hospitals adopting genomic surveillance face new ethical duties to disclose transmission events, while those that do not engage in such surveillance risk perpetuating inequities in patient safety and transparency. National guidance is urgently needed to ensure consistency in notification and promote ethical adoption of genomics. Coordinated frameworks from professional societies should define genetic thresholds warranting disclosure, address disparities in pathogen genomic detection methods, and uphold patient trust in the genomic era.

**Figure:**